# Atrial Cardiomyopathy Predicts Worse Outcome in Patients With Lung Cancer

**DOI:** 10.3389/fcvm.2022.932044

**Published:** 2022-07-01

**Authors:** Mengdi Ren, Yuyan Ma, Meng Wei, Yuye Ning, Hui Liu, Xue Shi, Yu Yao, Fengwei Guo

**Affiliations:** ^1^Department of Oncology, The First Affiliated Hospital of Xi’an Jiaotong University, Xi’an, China; ^2^Department of Neurology, The First Affiliated Hospital of Xi’an Jiaotong University, Xi’an, China; ^3^The Second Department of Neurology, Shaanxi Provincial People’s Hospital, Xi’an, China; ^4^Biobank, The First Affiliated Hospital of Xi’an Jiaotong University, Xi’an, China; ^5^Department of Cardiovascular Surgery, The First Affiliated Hospital of Xi’an Jiaotong University, Xi’an, China

**Keywords:** lung cancer, atrial cardiomyopathy, atrial fibrillation, oncocardiology, ischemic stroke

## Abstract

**Background:**

Reports of the clinical outcomes associated with the co-occurrence of atrial cardiomyopathy (ACM) and lung cancer (LC) are limited.

**Objectives:**

This study aims to investigate the influence of ACM on the prognosis of LC patients and related clinical determinants.

**Methods:**

Newly diagnosed LC patients from January 1st, 2015, to December 31st, 2020, were retrospectively enrolled at the First Affiliated Hospital of Xi’an Jiaotong University. The demographics and overall survival (OS) of the patients with or without ACM were compared. The survival rate was analyzed using the Kaplan–Meier method and multivariate Cox regression analysis. Binary logistic regression analysis was used to determine the risk factors for ACM.

**Results:**

A total of 306 patients (65.04 ± 10.30 years of age, 72.88% male) were analyzed. The prevalence of ACM in the non-small cell lung cancer (241, 78.76%) and small cell lung cancer (65, 21.24%) population was not statistically different. Overall, 53 (17.32%) LC patients had coexisting ACM. ACM patients were older (69 vs. 64, *p* = 0.0013) and had higher D-dimer levels (1.0 vs. 0.6, *p* = 0.001), lower serum calcium levels (2.23 vs. 2.31, *p* = 0.001), lower left ventricular ejection fraction (LVEF) values (67% vs. 69%, *p* = 0.036) and had more frequent coronary comorbidity disease (16.98% vs. 8.82%, *p* = 0.031). The median OS for patients with or without ACM was 15 months and 25 months, respectively (*p* = 0.018). Coexisting ACM compared to non-ACM was associated with worse OS in patients with LC (HR = 1.543, 95% CI: 1.042–2.283, *p* = 0.030).

**Conclusion:**

Coexisting ACM is associated with undesirable survival outcomes in patients with LC. These findings could help us to better understand the cardiac burden in these patients and provide additional risk stratification for them.

## Introduction

Lung cancer (LC) and cardiovascular disease (CVD) are two leading reasons of morbidity and mortality worldwide (1, 2). With the survival of LC patients greatly improved due to multiple revolutionary oncological treatments, such as immunotherapy and targeted-based therapy, several concomitant conditions that significantly impact survival, including CVD, and ischemic stroke (IS) (3, 4), have increased markedly (5, 6). LC and CVD share many risk factors that affect cancer-related survival (7, 8). A study showed that cancer patients are 2–6 times more likely to die of CVD or stroke than the general population (9).

Atrial cardiomyopathy (ACM), initially proposed more than a decade ago, is a pathophysiological concept describing covert atrial structural lesions and functions that involve architectural or physiological changes in the atria (10, 11). Previous research has considered ACM as one of the important etiologies of embolic stroke of undetermined source (ESUS), a subset of cryptogenic ischemic IS (12). The mechanisms of cryptogenic IS include occult structural cardiac lesion, hyper viscosity syndrome or undiagnosed cancer (13). Although it is widely acknowledged that there is a close association between IS and cancer (especially LC) (14), the mechanisms underlying the heightened risks of IS in cancer patients are still uncertain. Moreover, many ACM-related risk factors, such as advanced age, hypertension, diabetes, coronary heart disease, and chronic obstructive pulmonary disease (COPD) (15), are very common in LC patients (16, 17). In addition, cancer and anticancer therapy may cause pathological changes and directly affect atrial substrates (18). Therefore, it can be reasonably inferred that LC patients are also at high risk of ACM, which may confer a higher IS risk.

The influence of ACM on the prognosis of LC patients has never been studied. In this context, we hypothesized that coexisting ACM would be associated with an increased risk of IS and poor prognosis among general LC patients. We investigated the association of ACM with LC outcomes and further examined the related clinical parameters of ACM.

## Materials and Methods

### Study Population

**FIGURE 1:**
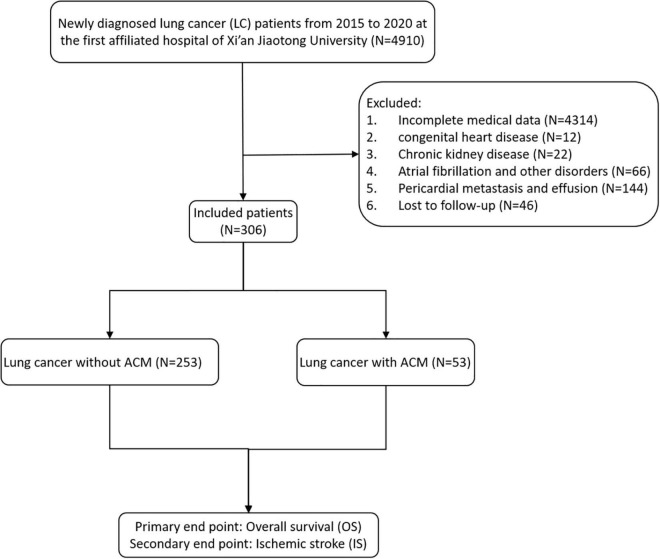
Flowchart detailing patient enrollment.

A total of 306 patients with newly diagnosed LC at the First Affiliated Hospital of Xi’an Jiaotong University between January 2015 and December 2020 were retrospectively enrolled in this study. The design of study is described in Figure 1. The protocol was carried out on the basis of the Declaration of Helsinki and approved by the Ethics Committee of the First Affiliated Hospital of Xi’an Jiaotong University (Approval No. XJTU1AF2021LSK-117). These patients were divided into two groups: LC patients with or without ACM. All clinical covariates were abstracted from electronic medical records.

### Diagnostic and Eligibility Criteria

ACM was defined as having at least one of the following biomarkers according to published literature: (1) *p*-wave terminal force in V1 (PTFV1) > 5,000 μV* ms; (2) N-terminal pro–B-type natriuretic peptide (NT-proBNP) > 250 pg/mL; and (3) severe left atrial enlargement (LAE: female > 38 mm; male > 40 mm) (19–21).

The inclusion criteria were as follows: (1) newly histologically or cytologically confirmed LC patients and (2) complete medical data for defining ACM and assessing cancer, namely, electrocardiogram (ECG), echocardiography, and NT-proBNP data.

Patients with any of the following conditions were excluded from this cohort: (1) cardiac and valvular history (congenital heart disease, other cardiomyopathies), myocardial infarction within 4 weeks, severe heart failure (left ventricular ejection fraction (LVEF) < 30%), intra-atrial thrombus, or infective endocarditis; (2) pericardial disease (pericardial metastasis, pericardial effusion); (3) heart arrhythmia disorder (atrial flutter, atrial fibrillation); and 4. renal insufficiency (serum creatinine ≥ 186 μmol/L or eGFR < 60 mL/min or chronic kidney disease (CKD) grade 3 and above).

### Statistical Analysis

Descriptive statistics are held up as the mean ± standard deviation (*SD*) for continuous normally distributed variables and as the median (25th percentile, 75th percentile) [*M* (QL, QU)] for non-normally distributed data. Categorical variables are presented as frequencies and percentages.

For continuous variables, the independent sample *t*-test is used to compare normally distributed data, and the Wilcoxon rank sum test was used to compare non-normal variables. Count data were statistically analyzed using the chi-square or Fisher exact test. Binary logistic regression was used to determine the risk factors for ACM. Overall survival (OS) was estimated by the Kaplan–Meier method, and significance was evaluated using the log-rank (Mantel–Cox) test. Mortality hazard ratios (HRs) were generated by multivariate Cox regression analysis using univariate Cox predictors. Statistical significance was defined as a *p* < 0.05.

## Results

### Patient Demographics

Our study included 306 patients; their median age was 65 years (range 29–94), and 72.88% were male. In all, 53 (17.32%) patients met the ACM diagnostic criteria. In Table 1, we present the baseline clinical characteristics of the LC patients with or without ACM. The distributions of sex, smoking history, pathological subtype, clinical stage, extrapulmonary/brain metastasis, hypertension, prior stroke, diabetes, and COPD were similar between the groups (*p*> 0.05), while those of age and coronary disease were different across the groups (*p*< 0.05). The ACM group had a slightly higher age (69.42 vs. 63.87) and more frequent coronary comorbidity disease (16.98% vs. 8.82%). We then analyzed the gene mutations and PD-L1 characteristics of the non-small-cell lung cancer (NSCLC) subgroup. The distributions of EGFR mutations/ALK fusions and PD-L1 expression levels were not related to ACM in the NSCLC patients (Table 2).

**TABLE 1:** Demographic characteristics of patients with LC.

Characteristic	All patients	LC with ACM	LC without AC	*P*
	(*N* = 306)	(*N* = 53)	(*N* = 253)	
Age at diagnosis, year	65.04 ± 10.30	69.06 ± 8.50	64.20 ± 10.46	0.001
Sex, female	83 (27.12)	13 (24.53)	70 (27.67)	0.640
Smoking, previous or current	207 (67.65)	37 (69.81)	170 (67.19)	0.711
Pathological subtype				
NSCLC	241 (78.76)	41 (77.36)	200 (79.05)	0.784
Adenocarcinoma	125 (40.85)	20 (37.74)	105 (41.50)	
Squamous cell	110 (35.95)	21 (39.62)	89 (35.18)	
Others	4 (1.31)		4 (1.58)	
Mixed	2 (0.65)		2 (0.79)	
SCLC	65 (21.24)	12 (22.64)	53 (20.95)	
Clinical stage^a^				0.165
Unknown	32 (10.46)	30 (11.86)	2 (3.77)	
Early	47 (15.36)	40 (15.81)	7 (13.21)	
Late	227 (74.18)	183 (72.33)	44 (83.02)	
Extra pulmonary metastasis				0.383
0	188 (61.44)	37 (69.81)	151 (59.68)	
1	76 (24.84)	10 (18.87)	66 (26.09)	
≥2	42 (13.73)	6 (11.32)	36 (14.23)	
Brain metastasis	27 (8.82)	4 (7.55)	23 (9.09)	0.482
Comorbidity disease history				
Hypertension	64 (20.92)	13 (24.53)	51 (20.16)	0.477
Coronary disease	27 (8.82)	9 (16.98)	18 (7.11)	0.031
Prior ischemic stroke	5 (1.63)	2 (3.77)	3 (1.19)	0.208
Diabetes	32 (10.46)	5 (9.43)	27 (10.67)	0.789
COPD	29 (9.48)	7 (13.21)	22 (8.70)	0.306
Treatment				
Chemotherapy	168 (54.90)	29 (54.72)	139 (54.94)	0.976
Radiation therapy	39 (12.75)	5 (9.43)	34 (13.44)	0.427
Surgery	33 (10.78)	9 (16.98)	24 (9.49)	0.110
Antiangiogenic treatment	26 (8.50)	5 (9.43)	21 (8.30)	0.787
Target therapy	47 (15.36)	8 (15.09)	39 (15.42)	0.953
Immunotherapy	57 (18.63)	6 (11.32)	51 (20.16)	0.133
Outcomes				
Ischemic stroke	12 (3.92)	1 (1.89)	11 (4.35)	0.699
Death	153 (50.00)	36 (67.92)	117 (46.25)	0.004
mOS; mo	24	15	25	0.018^b^

*Data are presented as the mean ± SD or No. (%).*

*ACM, atrial cardiomyopathy; NSCLC, non-small-cell lung cancer; SCLC, small-cell lung cancer; COPD, chronic obstructive pulmonary disease; mOs, median overall survival.*

*^a^Clinical stage: early stage, NSCLC stage I, II (TNM), SCLC limited stage; late stage, NSCLC stage III, IV (TNM), SCLC extensive stage; unknown: missing or not evaluated.*

*^b^Log-rank test.*

**TABLE 2:** Gene mutations and PD-L1 characteristics in the NSCLC subgroup.

	All patients	LC with ACM	LC without ACM	*P*
	(*N* = 241)	(*N* = 62)	(*N* = 195)	
Gene mutations, +	49 (20.33)	5 (11.63)	44 (22.22)	0.118
EGFR, +	39 (18.84)	5 (11.64)	34 (17.17)	
ALK, +	3 (1.45)	–	3 (1.52)	
Others, +	7 (3.38)	–	7 (3.54)	
PD-L1, +	25 (10.37)	3 (6.98)	22 (11.11)	0.584

*Data are presented as No. (%).*

*EGFR, epidermal growth factor receptor; ALK, anaplastic lymphoma kinase.*

*Gene mutations were recorded using fluorescent in situ hybridization (FISH) and immunohistochemistry in tissue microarray sections; PD-L1 IHC assays: SP263.*

### Atrial Cardiomyopathy in Non-small-Cell Lung Cancer vs. Small-Cell Lung Cancer Patients

Overall, the prevalence of ACM in the LC patients was 17.32% (Table 3). The prevalence of ACM in the NSCLC (241, 78.76%) and small-cell lung cancer (SCLC) (65, 21.24%) populations was not significantly different. The prevalence of ACM in the NSCLC and SCLC populations was not significantly different. In all the LC patients, the frequency of ACM was mostly due to the presence of NT-proBNP (94.34%) and a PTFV1 value > 5,000 μV⋅ms (9.43%). The prevalence of severe LAE was 7.55% in the ACM patients (Table 3).

**TABLE 3:** Prevalence of ACM in NSCLC vs. SCLC patients.

	All patients (*N* = 53)	NSCLC (*N* = 241)	SCLC (*N* = 65)	*P*
NT-proBNP > 250 pg/mL	50 (94.34)	40 (93.02)	12 (18.46)	0.712
PTFV1 ≥ 5,000	5 (9.43)	3 (6.98)	2 (16.67)	0.288
Severe large artery enlargement	4 (7.55)	4 (9.30)	0	0.582
ACM	53 (17.32)	43 (17.84)	12 (18.46)	0.854

*Data are presented as No. (%).*

*ACM, atrial cardiomyopathy; NSCLC, non-small-cell lung cancer; SCLC, small-cell lung cancer; NT-proBNP, N-terminal pronc-type natriuretic peptide; PTFV1, p-wave terminal force in V1.*

### Atrial Cardiomyopathy and Overall Survival

Half of the LC patients (153 of 306) died. To avoid deviation caused by different distributions of treatment methods, all the treatments that patients received after diagnosis were recorded (Table 1). The therapeutic mode was similar for patients with ACM compared to those without ACM, which implies that ACM is a risk factor for survival regardless of the subsequent treatment.

**FIGURE 2:**
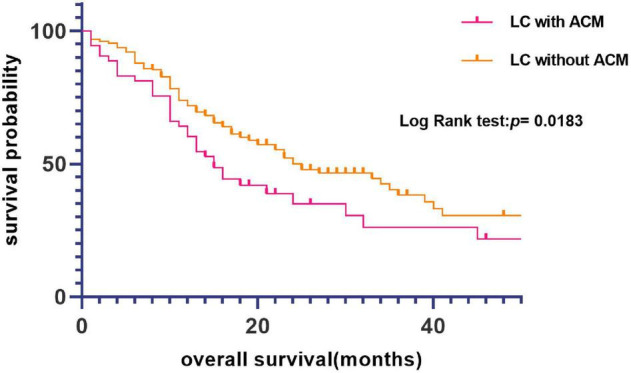
Kaplan–Meier curve of overall survival (OS) based on atrial cardiomyopathy (ACM).

To verify this, univariate analysis showed that sex, pathological subtype, clinical stage, smoking history, and ACM positivity had a significant association with survival (Table 4). Notably, Kaplan–Meier curves and log-rank tests showed that ACM was significantly associated with worse survival in LC patients (Figure 2). The median overall survival (mOS) for this LC patients was 23 months. The mOS for patients with or without ACM was 15 months and 25 months, respectively (*p* = 0.018) (Table 1).

**TABLE 4:** Univariate analyses of overall survival.

Characteristic	Chi-square	Log-rank *P*
Age (> 60 vs. ≤ 60)	1.94	0.164
Sex (male vs. female)	10.98	0.001
Pathological subtype (SCLC vs. NSCLC)	4.4	0.036
Clinical stage (late vs. early)	7.92	0.019
Smoking history (previous or current vs. never)	3.85	0.050
ACM (with vs. without)	4.8	0.018
Stroke (yes vs. no)	0.77	0.381
Extra-pulmonary metastasis (with vs. without)	2.15	0.341
Brain metastasis (with vs. without)	1.91	0.168

*ACM, atrial cardiomyopathy.*

In the multi-variable analysis of the Cox regression models, ACM was significantly associated with worse OS [hazard ratio (HR) = 1.556; 95% CI, 1.069–2.264; *p* = 0.021]. In the multivariate Cox proportional hazard model adjusting for clinicopathologic variables, the HR of the patients with ACM was 1.543 (95% CI, 1.042–2.283; *p* = 0.030) compared with that for the patients without ACM (Table 5), suggesting that ACM was an independent risk factor for LC patient prognosis.

**TABLE 5:** Multivariate Cox proportional hazard analyses of overall survival.

	Haz. ratio	*P*	[95% CI]
Sex (male vs. female)	2.684	0.004	1.369–5.264
Pathological subtype (SCLC vs. NSCLC)	1.588	0.015	1.092–2.308
Clinical stage (late vs. early)	2.336	0.003	1.337–4.081
Smoking history (previous or current vs. never)	0.755	0.340	0.424–1.345
ACM (with vs. without)	1.543	0.030	1.042–2.283

*ACM, atrial cardiomyopathy.*

### Risk Parameters Associated With Atrial Cardiomyopathy

Given that 18.5% of the LC patients had ACM and exhibited worse survival, we examined the risk factors in this group. The ECG, echocardiography, complete blood count (CBC) and blood biochemical examination tests performed closest to the date of diagnosis were used for the analysis. Our results showed that ACM patients had higher PTFV1 values (2,350 vs. 1,530, *p* < 0.000), higher NT-proBNP levels (460.1 vs. 68.54, *p* < 0.000), lower oxygen saturation values (95 vs. 96, *p* = 0.0299), higher pH (1.43 vs. 1.42, *p* = 0.0111), higher D-dimer levels (1.0 vs. 0.6, *p* = 0.0006) and prothrombin time (PT) (12.9 vs. 12.2, *p* = 0.0065), lower lymphocyte (1.17 vs. 1.28, *p* = 0.0469) and hemoglobin levels (123 vs. 133, *p* = 0.0049), higher CRP levels (mean CRP: 28.35 vs. 18.86, *p* = 0.00373), lower serum calcium levels (2.23 vs. 2.31, *p* = 0.001), and lower phosphate levels (0.96 vs. 1.03, *p* = 0.0046) (Table 6 and Supplementary Table 1).

**TABLE 6:** Comparison of baseline LC patients with/without ACM.

Parameter	LC with ACM (*n* = 53)	LC without ACM (*N* = 253)	*P*
PTVF1, μV*ms	2,350 (1,800–3,460)	1,530 (1,050–2,080)	0.000
NT-proBNP, pg/mL	460.1 (301.9–650.9)	68.54 (30.82–127.1)	0.000
SaO2,%	95 (93–96)	96 (95–97)	0.030
pH	7.43 (7.42–7.5)	7.42 (7.4–7.44)	0.011
D-D, mg/L	1 (0.5–2.5)	0.6 (0.3–1.1)	0.001
PT, s	12.9 (12–13.5)	12.2 (11.1–13.2)	0.007
ALC,10^9/L	1.17 (0.95–1.42)	1.28 (1.03–1.66)	0.047
HGB, g/L	123 (109–140)	133 (122–144)	0.005
CRP, mg/L	10 (10–41.7)	10 (10–14)	0.037
CK, U/L	54 (36–75.5)	63 (47–87)	0.048
ALB, g/L	35.2 (31.3–39)	39.65 (36.9–42.05)	0.000
Ca, mmol/L	2.23 (2.14–2.35)	2.31 (2.22–2.39)	0.001
P, mmol/L	0.96 (0.83–1.06)	1.03 (0.91–1.15)	0.005
LVEF,%	67 (63–71)	69 (65–73)	0.036
LVEDD, mm	49 (46–52)	47 (45–49)	0.005
LVESD, mm	30 (28–34)	28 (26–31)	0.002

*SaO_2_, oxygen saturation; D-D, D-dimer; PT, prothrombin time; ALC, absolute lymphocyte count; HGB, hemoglobin; CRP, C-reactive protein; CK, creatine kinase; ALB, albumin; LVEF, left ventricular systolic function; LVEDD, left ventricular end diastolic dimension; LVESD, left ventricular end systolic dimension.*

In addition, admission transthoracic echocardiograms were reviewed for all the patients. Those with ACM had lower LVEF values (67% vs. 69%, *p* = 0.0357). Univariate logistic analysis showed that patients with lower LVEF values were likely to be complicated with ACM (OR = 0.938, *p* = 0.007, 95% CI, 0.895–0.938). Hematological examinations and echocardiography indexes were included in the multivariate logistic regression analysis. As shown in Table 7, we found that higher D-dimer levels (*p* = 0.016, OR = 1.246) and lower serum calcium levels (*p* = 0.019, OR = 0.001) were significant risk factors for ACM. In the multivariate analysis, transthoracic echocardiogram indexes failed to show any meaningful value (Supplementary Table 2).

**TABLE 7:** Multivariate logistic regression analysis of clinical parameters.

Parameter	OR	*P*	95% conf. interval
D-D, mg/L	1.245	0.016	1.041–1.49
PT, s	1.477	0.149	0.869–2.509
ALC,10^9/L	1.068	0.916	0.312–3.661
HGB, g/L	0.967	0.056	0.934–1.001
CRP, mg/L	0.99	0.444	0.966–1.015
CK, U/L	0.999	0.953	0.988–1.011
ALB, g/L	1.02	0.774	0.89–1.168
Ca, mmol/L	0.001	0.019	0.000–0.316
P, mmol/L	0.099	0.174	0.004–2.776

*SaO_2_, oxygen saturation; D-D, D-dimer; PT, prothrombin time; ALC, absolute lymphocyte count; HGB, hemoglobin; CRP, C-reactive protein; CK, creatine kinase; ALB, albumin.*

## Discussion

In this observational, retrospective cohort study, 585 patients with LC were followed for a median of 20 months. In our study, the prevalence of LC patients with ACM was 17.32%, and these patients had a higher age (69 vs. 65) and more frequent coronary comorbidity disease (16.98% vs. 8.82%). However, the prevalence of ACM in LC patients was not significantly different in different pathological subtypes. In addition, we found that coexisting ACM was associated with worse OS (15 vs. 25) in patients with LC, who had higher D-dimer levels, lower serum calcium levels and lower LVEF values than the non-ACM patients. Together, these findings support that the comorbidity of ACM is associated with poorer survival in LC patients. However, we did not find that cancer-related IS was associated with ACM. These data highlight the need for further studies to better investigate the underlying mechanisms of stroke and cancer.

### Atrial Cardiomyopathy Is Prevalent in Lung Cancer Patients

Any abnormalities of the atria of structural, architectural, contractile, or electrophysiological changes have been used to define a new entity known as ACM (22). In EHRAS (European Heart Rhythm Association; EHRA/Heart Rhythm Society; HRS/Asian Pacific Heart Rhythm Association; APHRS/Latin American Society of Electrophysiology and Cardiac Stimulation; SOLAECE) classification, ACM were defined into four types: principal cardiomyocyte changes; fibrotic changes; combined cardiomyocyte-pathology/fibrosis and non-collagen infiltration (23). However, most patients would not receive myocardial biopsy, making histological and pathopysiological classification hard to evaluate. At present, there is no absolute diagnostic criteria for ACM, but most of the studies are defined by the relevant markers of ACM. For example, it is reported that PTFV 1 abnormality is related to the increase of left atrial volume and the decrease of left atrial emptying fraction and reservoir function (24). Any pathological state that causes atrial dysfunction can lead to the increase of PTFV1, implying this ECG biomarkers may be the signs of ACM. Numerous randomized studies have adopted the combined biomarkers to define ACM (25). Other biomarkers includes LAE, paroxysmal supraventricular tachycardia, bayes syndrome, and serum biomarkers associated with atrial dysfunction, etc. In recent years, more emerging imaging techniques (such as cardiac computed tomography or magnetic resonance imaging and so on) have been used for accurate assessment of atrial structure and function, which may provide more supports of detecting ACM (26, 27). A study use late gadolinium enhancement MRI (LGE-MRI) to evaluate atrial fibrosis and it is associated with appendage thrombus (28).

A previous study reported that the prevalence of ACM (26.6%) is increased in patients with ESUS compared to patients with other established etiologies for IS (21). In this study, we found that 17.32% of the LC patients had ACM. The key clinical determinants of ACM are unclear, but it is reported to be related to many disease or conditions, such as aging, smoking, hypertension, diabetes, coronary heart disease, and heart failure (15, 29). These high-risk factors overlap in most LC patients (30). This is also shown in this study. In addition, ACM-related biomarkers play an important role in the development of malignancies (18), suggesting that some physiological changes are shared by LC and ACM. For example, various studies have confirmed that NT-proBNP, a biomarker of ACM, and other biomarkers of CVD have prognostic significance in cancer (31, 32). A study showed that coexisting CVD precancerous polyps lead to tumor progression and secretion of cardiac excreted factors, mechanically supporting the above hypothesis (33).

On the other hand, recognition of the interaction between cancer and atrial fibrillation (AF) has shed new light on the relationship with ACM. The basic pathology characteristic of AF and ACM is myocardial fibrosis. Fibrosis can be caused by inflammation and its mediators (34), such as systemic infection and autoimmune diseases, and may also occur in chronic inflammation (such as cancer) (35). Atrial fibrosis can precede AF or even exist without AF, which implies that ACM is the substrate for AF (18). This means that cancer or other coexisting subclinical inflammatory diseases (such as hypertension and coronary artery disease) and conditions (like aging and endocrine abnormalities) can produce inflammatory mediators, which change atrial electrophysiology and structural substrates (36). Many studies have reported that patients with malignancy have increased susceptibility to AF (37, 38), which also supports this theory.

### Atrial Cardiomyopathy Predicts Worse Survival

We explored the relationship between ACM and LC and its influence on the survival of LC patients. The coexisting ACM was significantly associated with worse survival in patients with LC.

The biological mechanisms by which ACM may influence prognosis are unclear, but several lines of evidence suggest that this result is biologically reasonable. First, cancer is a systemic inflammatory condition originated from a combination of genetic, habitual and environmental factors (39). ACM has been associated with several clinical comorbidities and inflammatory conditions, such as systemic infections (15, 34). This means that the various comorbidities or conditions that may adversely affect the occurrence and outcomes of LC could also lead to ACM. Second, the factors that cause abnormalities in atrial tissue substrates can also be systemic manifestations of tumor progression. Cancer can promote the development of atrial fibrosis, leading to metabolic and electrolyte abnormalities, fluid imbalance, and infections. These inflammatory states then contribute to atrial remodeling (40), making ACM a prognostic marker. In other words, there is a bidirectional and progressive relationship between ACM and LC. The abnormality of many ACM-related markers has been shown to be associated with the prognosis of cancer patients. For example, NT-proBNP levels are related to the severity of malignancy without cardiac disease or cardiotoxicity in anticancer therapy (41, 42). In addition, atrial lesions have substantial related adverse outcomes including arrhythmogenic changes, atrial fibroblast proliferation, hyperinnervation, and thrombogenic changes (23, 43), all of which can lead to worse prognosis in lung cancer patients. For instance, arterial thrombosis is a marker of occult cancer (especially lung cancer) and an unfavorable prognostic factor (44, 45). Taken together, with the effect of cancer and other risk factors, pathological changes of atrial cells (like myocardial hypertrophy, fibrosis, and fatty infiltration and so on) results in mechanical dysfunction or abnormal electrical conduction, which eventually converts to atrial dilatation and the congestive heart failure. These factors all contributes to the worse survival of LC. Our study showed that patients with lower LVEF values were likely to be complicated with ACM, which buttressed that view.

### Atrial Cardiomyopathy Is Not Related to Ischemic Stroke Among Lung Cancer Patients

A new perspective of the relationship between AF and stroke has emerged in the past decade.

Although AF has been proved to be related to IS, the causal relationship between them is still indirect. A study found that there was no consistent time correlation between AF and IS (46).

A study reported that variants of chromosome associated with increased risk of cardioembolic IS, even in those not detected to have AF (47). A study reported nearly 65% of patients with cryptogenic stroke have ACM (20). These evidences verify that ACM may be the basis of IS.

Taken together, ACM is an important marker of increased risk of thromboembolism, particularly IS. Atrial abnormality forms the substrate for thrombus formation and AF may be a sign of potential risk of ACM. ACM itself, even without AF, is a risk factor for stroke (48). The mechanism of elevated risk of thrombus formation is likely related to the interaction between a generalized and local pro-thrombotic and inflammatory state (22). Moreover, atrial fibrosis, enlargement, and dysfunction may further lead to atrial congestion, pre-thrombotic state and subsequent IS (49).

Clinical trials have shown that treatment with ACM may reduce the risk of IS (50). Cryptogenic stroke (40–51%) is more common in cancer patients than in the general population (51). Compared with cancer-free controls, survivors of LC show a higher risk of stroke (52). Previous studies have reported that 45–65% of cryptogenic stroke patients have comorbidities of ACM (19, 20). Therefore, there are good reasons to hypothesize that ACM has a strong association with cancer-related stroke.

Unfortunately, we have not demonstrated this relevance in this set of data. The univariate analysis showed that there was no significant correlation between stroke and survival (Table 4). There are several possible reasons. The main reason is that the number of stroke events was too small in this cohort to verify the conclusion. Some patients received treatments at other centers, leading to some lost-to-follow-up and thus unknown ending events. Moreover, in order to evaluate the comorbidity of ACM, many patients with incomplete information occurring ending events were excluded from analysis. However, given that ACM is associated with a second era in understanding the relationship of disorders of the atria to stroke risk and anticoagulant therapy is still open to debate, we look forward to more research in this field in the future to reveal the etiology of stroke in cancer patients.

### Study Limitations

Our study has limitations due to its single-center design, small sample size, and incomplete matching or exclusion of many patients from the analysis. Second, data were extract retrospectively, and much clinical information related to tumor assessment was not complete and lacked elaboration. Third, this study failed to analyze the impact of completing treatment for some patients who received treatments at other centers. More studies are needed in the future, such as prospective studies to determine reliable biomarkers to predict cancer-related stroke and clinical trials to determine the treatment and prevention of ACM.

## Conclusion

Our study provides the first evidence that the comorbidity of ACM predicts worse prognosis in patients with LC. In addition, we found that higher D-dimer levels, lower serum calcium levels, and lower LVEF values were significant risk factors for ACM. NT-proBNP and PTFV1 are not routine clinical assessments for cancer patients. Our results imply that patients with those abnormal indexes may have coexisting ACM and a worse prognosis. Given that patients with ACM have a higher risk of poor survival, more frequent follow-up and detection in these patients should be considered.

## Data Availability Statement

The raw data supporting the conclusions of this article will be made available by the authors, without undue reservation.

## Ethics Statement

The studies involving human participants were reviewed and approved by the Ethics Committee of the First Affiliated Hospital of Xi’an Jiaotong University. The patients/participants provided their written informed consent to participate in this study.

## Author Contributions

MR and YM contributed to clinical data collection and analysis. HL and XS provided support and assistance in data extraction. All authors contributed to the article and approved the submitted version.

## Conflict of Interest

The authors declare that the research was conducted in the absence of any commercial or financial relationships that could be construed as a potential conflict of interest.

## Publisher’s Note

All claims expressed in this article are solely those of the authors and do not necessarily represent those of their affiliated organizations, or those of the publisher, the editors and the reviewers. Any product that may be evaluated in this article, or claim that may be made by its manufacturer, is not guaranteed or endorsed by the publisher.
