# Supplementary material for: Atrial Cardiomyopathy Predicts Worse Outcome in Patients With Lung Cancer
Source: Front Cardiovasc Med. 2022 Jul 1;9:932044. doi: 10.3389/fcvm.2022.932044 (PMC9283710; doi:10.3389/fcvm.2022.932044)
Supplement: Supplementary file 1 [file Data_Sheet_1.docx]

Supplementary Material

**Supplementary Table 1:** Comparing baseline of Lung cancer (LC) patients with/without ACM

| Parameters | LC With ACM (N= 53) | LC without ACM (N=253) | P Value |
| --- | --- | --- | --- |
| P-R, ms | 148(132-170) | 152(140-168) | 0.568 |
| mLactate, mmol/L | 1.5(1.2-2.6) | 1.4(1-1.8) | 0.361 |
| NSE, ng/ml | 21.115(15.57 -31.02) | 19.7(15.9-27.52) | 0.474 |
| CA-199, U/ml | 10.93(7.44-25.49) | 12.28(6.79-22.36) | 0.593 |
| CA-125, U/ml | 27(13.8-43.8) | 20.4(11.8-61.9) | 0.430 |
| CEA, ng/ml | 2.44(2.19-3.58) | 2.765(1.99-3.71) | 0.907 |
| CYFRA21-1, ng/ml | 4.975(2.53-11.87) | 3.84(2.61-8.13) | 0.292 |
| FDP, mg/L | 3.08(2.3-6.73) | 3.5(2.65 -5.08) | 0.682 |
| TT, s | 16.6(16.1-17.1) | 16.7(16.1-17.2) | 0.366 |
| APTT, s | 30.8(27.1-35.6) | 29.2(26.6-35.35) | 0.444 |
| ANC,10^9/L | 4.13(3.4-6.2) | 4.68(3.41-6.06) | 0.858 |
| WBC,10^9/L | 6.175(4.89 -7.89) | 6.065(3.75-8.15) | 0.465 |
| PLT,10^9/L | 237(189-296) | 241(192-294) | 0.749 |
| ESR, mm/h | 18(15-65) | 22(11-45) | 0.531 |
| CKMB, U/L | 11.05(7.5-16) | 10.5(7-14) | 0.263 |
| HBDH, U/L | 168(148-206.5) | 162(138-193) | 0.133 |
| LDH, U/L | 224.5(194-297.5) | 216.5(187 -255) | 0.372 |
| GLO, g/L | 28.7(24.9-30.6) | 27.8(25.1-30.8) | 0.860 |
| Mg, mmol/L | 0.964±0.078 | 0.974±0.094 | 0.475 |
| Cl, mmol/L | 101(96.1-103.9) | 101.1(98.4-103.7) | 0.309 |
| Na, mmol/L | 140(135-142) | 140(138-142) | 0.625 |
| K, mmol/L | 4.04(3.71- 4.34) | 4.1(3.9-4.37) | 0.161 |
| IST, mm | 8(8-9) | 8(8-9) | 0.492 |
| ISPR, mm | 8(8-9) | 8(7-9) | 0.510 |
| IVPWT, mm | 8(8-8) | 8(8-8) | 0.189 |
| LVPWPR, mm | 10(10-11) | 10(10-11) | 0.617 |

Data are presented as mean± SD or median (QL, QU).

Abbreviations: ACM: atrial cardiomyopathy; NSE=neuron-specific enolase; CEA=carcinoembryonic antigen; FDP= Fibrinogen and Fibrin Degradation Products; APTT=active partial Thrombin time; TT=Thrombin time; ANC=absolute neutrophil count; WBC=white blood cell; PLT= Platelet; ESR=erythrocyte sedimentation rate; CKMB=Creatine Kinase Myocardial Band; HBDH=hydroxybutyrate-dehydrogenase; LDH=lactate dehydrogenase; IST=interventricular septal thickness; ISPR= interventricular septal pulsatile range; IVPWT= left ventricular posterior wall thickness; LVPWPR=left ventricular posterior wall pulsatile range**.**

**Supplementary Table 2:** Multivariate logistic regression analysis of transthoracic echocardiography (TTE)

| Parameters | OR | P value | 95% Conf. Interval |
| --- | --- | --- | --- |
| LVEF, % | 1.013 | 0.892 | 0.835-1.230 |
| LVEDD, mm | 1.006 | 0.968 | 0.736-1.375 |
| LVESD, mm | 1.15 | 0.574 | 0.706-1.873 |
| IST, mm | 1.055 | 0.763 | 0.745-1.493 |
| ISPR, mm | 1.031 | 0.826 | 0.787-1.351 |
| IVPWT, mm | 1.275 | 0.422 | 0.705-2.308 |
| LVPWPR, mm | 0.949 | 0.753 | 0.684-1.316 |

Abbreviations: LVEF=left ventricular systolic function; LVEDD= left ventricular end diastolic dimension; LVESD= left ventricular end systolic dimension; IST=interventricular septal thickness; ISPR= interventricular septal pulsatile range; IVPWT= left ventricular posterior wall thickness; LVPWPR=left ventricular posterior wall pulsatile range.
